# GPT-based prediction of short-term survival following decompressive hemicraniectomy in malignant middle cerebral artery infarction

**DOI:** 10.3389/fneur.2025.1603536

**Published:** 2025-07-24

**Authors:** Sebastian Lehmann, Martin Vychopen, Erdem Güresir, Johannes Wach

**Affiliations:** Department of Neurosurgery, University Hospital Leipzig, Leipzig, Germany

**Keywords:** decompressive hemicraniectomy, middle cerebral artery infarction, artificial intelligence, GPT, survival, functional outcome, prediction

## Abstract

**Introduction:**

An analysis of the prognostic ability of the large language model (LLM) Generative Pre-trained Transformer (GPT) to predict short-term survival and functional outcomes in patients with malignant middle cerebral artery (MCA) infarction following decompressive hemicraniectomy.

**Methods:**

This retrospective study included 100 patients with malignant MCA infarction who underwent decompressive craniectomy (DC). GPT-4 and GPT-4 Omni were used to predict patient outcomes based on 20 patient-specific factors. Each version of GPT was tested with and without context enrichment (CE). CE versions were provided with the current AHA/ASA 2019 guidelines and meta-analyses of RCTs to inform decision-making. The real-life outcome of the patients, measured by the modified Rankin Scale (mRS), served as a reference. The following endpoints were evaluated: survival during inpatient stay, achievement of a functional status of mRS 0–4 at discharge, and at 3-, 6-, and 12-months post-discharge. We analyzed the prognostic prediction of GPT by calculating the area under the curve (AUC) and determining the optimal cutoff using the Youden index for divergent prediction outcomes. After dichotomization according to the cutoff set, a chi-squared test (two-sided) was performed.

**Results:**

GPT-4 and GPT-4 Omni demonstrated the ability to estimate survival during in-hospital stay. In both versions, the CE GPT outperformed the non-CE versions. GPT-4 Omni (CE) achieved an AUC of 0.67 (95% CI: 0.54–0.79; *p* = 0.002), while GPT-4 (CE) reached an AUC of 0.70 (95% CI: 0.57–0.82; *p* = 0.018). GPT-4 also achieved statistical significance even without CE (AUC of 0.66; 95% CI: 0.53–0.78; *p* = 0.018). In contrast, the non-CE version of GPT-4 Omni did not reach significance in predicting the survival of hospitalization (AUC of 0.60; 95% CI: 0.48–0.73; *p* = 0.07). For questions regarding the functional outcome of patients, neither version of GPT was able to make a sufficient prognostic prediction. However, when provided with the pre-stroke mRS, GPT-4 Omni was able to predict the mRS at discharge (*p* = 0.01; Pearson's correlation coefficient = 0.696).

**Conclusion:**

The study shows the already existing high potential of AI in predicting short-term outcomes. It also shows the existing limitations for the evaluation of more complex questions, such as functional outcomes.

## Introduction

The use of artificial intelligence (AI) is becoming increasingly important for medical use. Regarding prognostic abilities, there are already publications suggesting that AI-based image morphological recognition of stroke extent has potential comparable to that of an experienced neuroanatomist (1). In the significantly more complex detection of acute common and severe diseases based on clinical data, Levine et al. (1) demonstrated that Chat GPT 3 outperformed non-medically trained individuals, but not physicians. Bentley et al. (2) used machine-learning-based image recognition software to predict hemorrhagic transformation after intravenous thrombolysis in ischemic stroke. Using supervised machine learning algorithms, another research group was able to predict the outcome of patients with ischemic stroke after intra-arterial therapy with an accuracy of ~70% (3). Despite these promising results, GPT has not yet been shown to predict the 6-month outcome after traumatic brain injury due to insufficient specificity (4). However, promising results have been reported regarding GPT's potential for outcome prediction in aneurysmal subarachnoid hemorrhage (5).

MCA infarction is a severe condition with high mortality and a major impact on the patient's quality of life in cases of survival. In malignant MCA Infarction, decompressive hemicraniectomy is the ultima ratio for preserving the patient's life (6). To date, the prediction of the prognosis for these patients remains extremely difficult.

The functionality of modern AI is based on complex digital neural networks, which are created based on real data and are capable of processing complex tasks using deep learning techniques (7). One of the most advanced AI-based applications currently available for public use is GPT. GPT is a language model that was developed and trained by the company OpenAI to generate answers that are as human-like as possible (8). GPT processes data and relates them to each other within a network and creates a so-called “transformer architecture” to enable precise categorization within the respective context (8, 9).

The present study is the first to investigate GPT's current ability to process complex real-life patient data into prognostic estimation of the patient's clinical outcome.

## Materials and methods

This retrospective analysis investigated the capability of deriving a prognosis using a single data modality input from patient data. Data were collected from patients admitted to the hospital with malignant MCA infarction who underwent emergency decompressive hemicraniectomy. To further enhance prognostic assessment, the study investigated whether providing context for decision-making can contribute to improving predictive accuracy. Data from 100 patients who underwent decompressive hemicraniectomy for MCA infarction at Leipzig University Hospital between 2016 and 2023 were assessed. To provide the large language model (LLM) with comprehensive input, patient-specific parameters (age, gender, previous cardiac diseases, intake of blood-thinning medication, laboratory parameters such as leukocytes, platelets, CRP, and preoperative pTT), disease-specific parameters [infarct size, hemorrhagic transformation, pupil status, mRS, and Glasgow Coma Scale (GCS)], and therapy-specific parameters (volume and diameter of the decompression and hemoglobin levels before and after the procedure) were sampled and provided to the AI anonymously. These parameters have largely already been associated with the prognosis after decompressive hemicraniectomy in previous studies (10–15).

The infarct volume was calculated using the Brainlab Suite's volumetric function (Brainlab, Feldkirchen, Germany) (16). To depict the extent of the decompressive hemicraniectomy, both the AP diameter usually given in the literature and the surface area of the decompressed area were specified according to the formula As = π[(d/2)^2^ + h^2^] (17).

The neurological outcome of the patients was assessed using the modified Rankin Scale (mRS) (18). In accordance with the existing prospective randomized studies—DECIMAL (19), HAMLET (20), DESTINY (21), and DESTINY II (22)—the mRS was also included in the analysis at the time of discharge, and after 3 months, 6 months, and 1 year.

The data were provided to ChatGPT in a standardized chat prompt. For our investigation, we utilized two versions of GPT: GPT-4, released in March 2023, and the advanced version, GPT-4 Omni, released in May 2024 (23). A total of five questions were formulated, each of which had to be answered with a yes/no response. Each chat prompt was offered to both versions of GPT with and without context-enrichment (CE) to provide the LLM a defined base for reasoning. As CE, we chose the current 2019 ASA/AHA guideline (6) as well as a meta-analysis of the prospective randomized studies (24) in patients under 60 years of age, and the prospective randomized study DESTINY II (22) in patients over 60 years of age. Each question was asked a total of 3 times to consider divergent answers. The mean of the given answers was documented. The answers were scored as follows: three times “no” (score: 0), two times “no” (score: 0.33), one time “no” (score: 0.66), and three times “yes” (score: 1.00). GPT was asked to evaluate the survival during the in-hospital stay, as well as the functional outcome at discharge, 3, 6, and 12 months in a yes or no answer. Favorable (mRS 0–4) and non-favorable outcomes (mRS 5–6) were dichotomized as defined in the prospective randomized studies (24). An exemplary chat prompt is provided in Supplementary Figures 1, 2.

**Figure 1:**
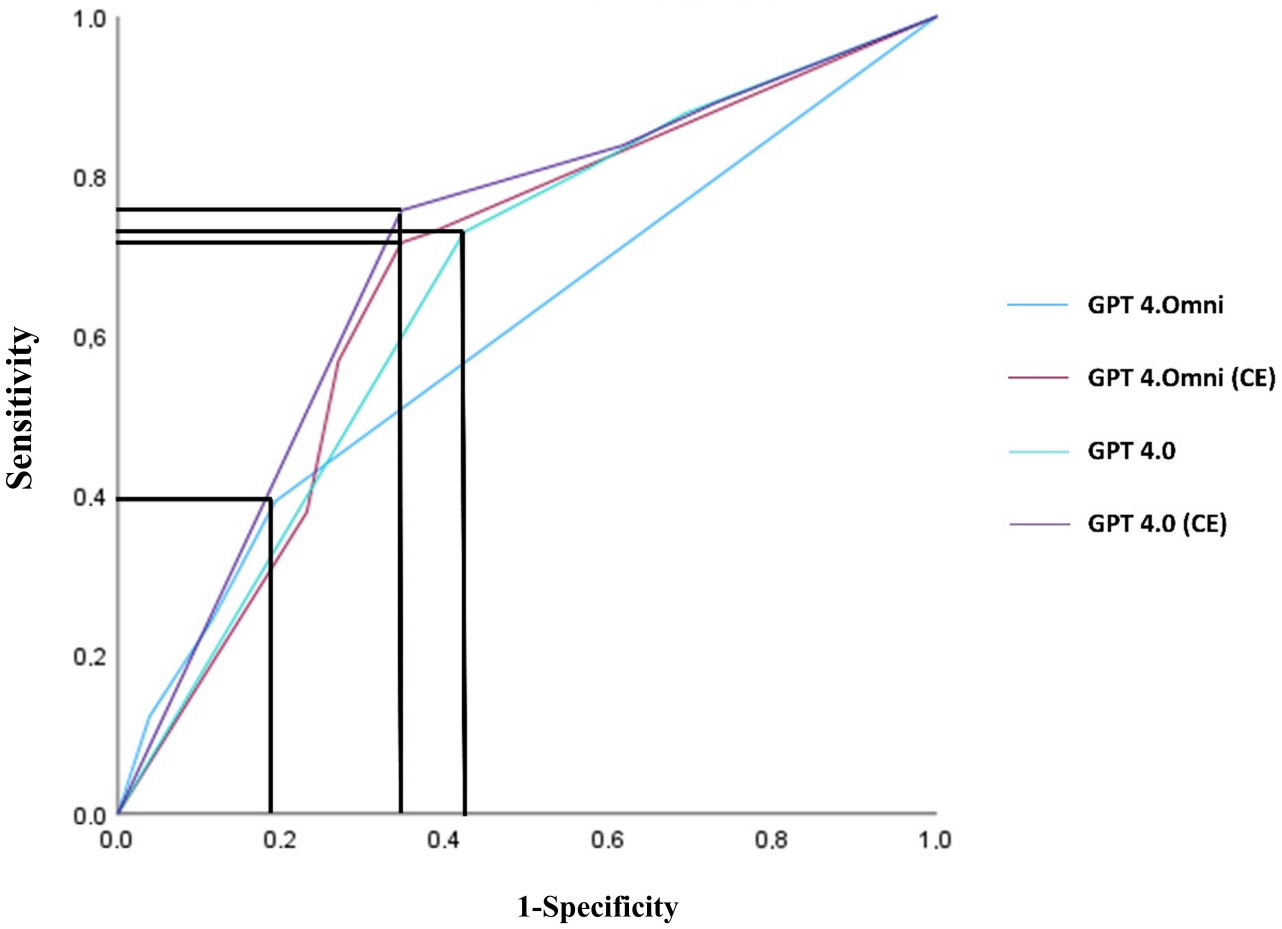
ROC analysis of the response variability of the multiple responses for survival at discharge. The line indicates the highest Youden index.

Data were entered into an anonymized database, and this database was analyzed with SPSS (IBM Corp., Released 2023. IBM SPSS Statistics for Windows, Version 29.0.2.0, Armonk, NY: IBM Corp). First, we performed a the descriptive analysis of our cohort (Table 1). GPT's answers were subjected to a receiver operating characteristic analysis (ROC) to determine the area under the curve (AUC), sensitivity, and specificity stated with the 95% confidence interval (CI) (Figure 1). The Youden index was calculated to define the optimal cutoff in the case of divergent answers (Table 2). After dichotomization according to the determined cutoff, GPT's answers were tested for significance using a chi-squared test.

**Table 1:** Comparison of the Leipzig cohort with the cohorts of the randomized studies DECIMAL, DESTINY I/II, and HAMLET.

**Cohort**	**Age**	**Male**	**Death at one year**
Study group Leipzig	59.3	68%	39.8%
Subgroup ≤ 60 years	52	72.4%	27.1%
DECIMAL (surgery group)	43.5	45%	20.0%
DESTINY (surgery group)	43.7	47%	17.6%
HAMLET (surgery group)	50	63%	22%
Subgroup ≥61 years	68.3	59.6%	55%
DESTINY II (surgery group)	70	51%	43%

**Table 2:** Results of the ROC analyses of divergent answers for survival at discharge (Question 1) for the different versions of GPT, shown with 95% CI and asymptotic significance level and the optimal cutoff for dichotmisation determined by the highest Youden Index highlighted in bold.

**Question 1**	**AUC**	**Significance**	**95% CI upper limit**	**95% CI bottom limit**	**Highest youden index**	**Determined cutoff**	**Sensitivity at cutoff**	**Specificity at cutoff**
GPT-4 Omni	0.60	0.120	0.48	0.72	0.20	**0.17**	0.39	0.8080
GPT-4 Omni (CE)	0.67	0.010	0.54	0.80	0.37	**0.32**	0.72	0.65
GPT-4	0.66	0.016	0.53	0.79	0.31	**0.83**	0.73	0.58
GPT-4 (CE)	0.70	0.002	0.58	0.821	0.411	**0.83**	0.76	0.65

**Figure 2:**
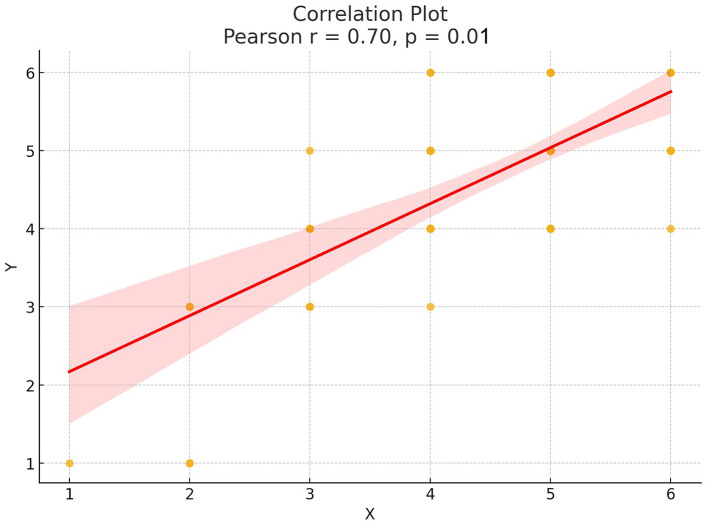
Scatterplot for GPT-4 Omni's responses to the question about mRS at discharge, given pre-stroke mRS vs. real outcomes.

In an additional prompt, the pre-stroke mRS was included to refine the mode (Supplementary Figure 4). GPT-4 could not be included in the following analysis as it had been replaced by OpenAI with a more recent version. GPT-4 Omni was asked to predict the mRS at the time of discharge. A delta (Δ) between the pre-stroke mRS and the mRS at the time of discharge was calculated for GPT's estimation, as well as the real mRS (Figure 2). Subsequently, the ΔmRS was assessed by Pearson's correlation coefficient.

## Results

### Patient characteristics

In our patient cohort, 68% were male, with a median age of 59 years. The median GCS score prior to surgery was 10. According to the parameters in the HAMLET, DESTINY, DESTINY II, and DECIMAL studies, the cohort was divided into patients aged over 61 years and those aged ≤ 60 years. In the younger cohort, the median age of onset was 53 years, and 72.4% were male (19–22, 24). Among patients 61 years or older, the median age was 68 years, with 59.6% male patients. The 1-year mortality rate across all ages was 39.8%. Of these, the cohort of >61-year-olds accounted for the largest proportion, with 55% of patients dying after 1 year (Table 1).

### GPT's performance in the estimation of survival

During the 3-fold presentation of each individual patient to GPT, it was shown that GPT could show divergent answers to the same question, regardless of the version used. For the analysis of survival estimation at the time of discharge, the rate of divergent answers varied from 18 to 30%, with GPT-4.0 showing less divergence than GPT-4Omni [GPT-4.0 18%, GPT-4.0 (CE) 20%, GPT-4 Omni 24%, and GPT-4 Omni (CE) 30%].

In the ROC-analysis (Figure 1) to determine the optimal cutoff for a positive answer in cases of divergent answers regarding survival at discharge, the highest Youden index was achieved with ≥2 positive answers for GPT-4 (>0.66), and ≥1 positive answer for GPT-4 Omni (>0.33). The AUC values of the LLMs ranged from 0.60 to 0.70, with the CE versions outperforming the non-CE-GPT versions in the overall analysis (AUC: GPT-4 Omni non-CE = 0.60, GPT-4 Omni CE = 0.67; GPT-4 non-CE = 0.66, GPT-4 CE = 0.70). In the subgroup analysis, GPT-4 showed weaker results in patients ≥61 years, where GPT-4 Omni outperformed both CE and non-CE versions. GPT-4 CE performed the worst (AUC: GPT-4. Omni non-CE = 0.61, GPT-4 Omni CE=0.68; GPT-4 non-CE = 0.61, GPT-4 CE = 0.59). Significant diagnostic correlations between survival at time of discharge and the estimations of GPT-4 Omni (CE) (*p* = 0.01, 95% CI 0.54–0.79), GPT-4 (CE) (*p* = 0.002, 95% CI 0.57–0.82), and non-CE (*p* = 0.016, 95% CI 0.53–0.78) were observed (Table 2). The answers were dichotomized according to the cutoff set by the Youden index (Table 2). According to the highest Youden index calculated based on the ROC curve analysis, the cutoff for GPT-4 was set at ≥1/3 positive answers, and for GPT-4 Omni at 2/3 positive answers. Subsequently, GPT's prognoses were compared to real outcomes using cross-tabulation. In the chi-squared test for survival during in-hospital stay (Question 1), GPT significantly predicted patient survival with GPT-4 Omni (CE) (*p* = 0.002), GPT-4 (CE) (*p* = 0.018), and non-CE (*p* = 0.018). GPT4 Omni non-CE narrowly missed statistical significance (*p* = 0.07) and showed considerably reduced sensitivity (Table 3).

**Table 3:** Cross-table depiction of GPT's answers compared to real outcome after cutoff-based dichotomization; The first value describes the prognosis by GPT, the second value represents the real outcome; Chi-squared test and *p-value* for survival (mRS <6) at discharge A: GPT-4 Omni, B: GPT-4 Omni with context enrichment (CE), C: GPT-4, D: GPT-4 with context enrichment (CE).

**GPT-4 Omni** **cutoff 1/3 answers**	**GPT survival at discharge**	**GPT no survival discharge**	**p-value**
**A**			
mRS 6	21/26 (81%)	5/26 (19%)	0.07
mRS 0–5	45/74 (61%)	29/74 (39%)	
Total	66/100	34/100	
**GPT-4 Omni (CE) cutoff 1/3 answers**	**GPT survival at discharge**	**GPT no survival discharge**	**p-value**
**B**			
mRS 6	16/26 (62%)	10/26 (38%)	0.002
mRS 0–5	20/74 (27%)	54/74 (73%)	
Total	36/100	64/100	
**GPT-4 cutoff 2/3 answers**	**GPT survival at discharge**	**GPT no survival discharge**	**p-value**
**C**			
mRS 6	11/26 (42%)	15/26 (58%)	0.018
mRS 0–5	14/74 (19%)	60/74 (81%)	
Total	36/100	64/100	
**GPT-4 (CE) cutoff 2/3 answers**	**GPT survival at discharge**	**GPT no survival discharge**	**p-value**
**D**			
mRS 6	10/26 (38%)	16/26 (62%)	0.018
mRS 0–5	12/74 (16%)	62/74 (84%)	
Total	22/100	78/100	

In the subgroup analyses regarding the prognosis for survival at discharge in groups ≥61-year-old patients and <61-year-old patients, GPT-4 Omni (CE) achieved significance for both groups (≥61 years, *p* = 0.014; < 61 years, *p* = 0.034). For the other models, only GPT-4 reached significance (*p* = 0.036) in ≥ 61-year-olds (Supplementary Figure 3, Supplementary Tables 1–3).

### GPT's performance in the estimation of functional outcomes

For the questions on the functional outcome (Questions 2–5), GPT provided almost exclusively negative answers (87%−100%). Resulting from ROC curve analysis and Youden index calculation, the cutoff was set to 2/3 positive answers for GPT-4 and 3/3 positive answers for GPT-4 Omni (Supplementary Figure 5, Supplementary Table 4). There was no significance for any of the questions across all tested GPT versions with and without CE, with only minimal differences between the versions and questions (Supplementary Table 5).

The prompt including the pre-stroke mRS, provided to GPT-4 Omni, resulted in usable mRS estimations at the time of discharge by the LLM. Pearson's correlation coefficient showed a significant correlation (*p* = 0.01) with a strong to very strong positive correlation (Pearson's correlation coefficient: 0.696, Figure 2, Table 4).

**Table 4:** Pearson's correlation coefficient of mRS estimation given the pre-stroke mRS for GPT-4 Omni.

		**Delta_mRS(GPT-4 Omni)**	**Delta_mRSReality**
**Correlation**			
Delta_mRS (GPT-4 Omni)	Pearson's correlation coefficient	1	0.696
	Sig. (two-sided		<0.001
	*N*	100	100
Delta_mRSReal	Pearson's correlation coefficient	0.696	1
	Sig. (two-sided)	<0.001	
	*N*	100	100

The calculation was based on the delta of the mRS from the time points of pre-stroke to discharge.

## Discussion

Our study shows that GPT can estimate prognosis for patients with malignant infarcts of the middle cerebral artery who have undergone decompressive hemicraniectomy based on patient profiles.

After dividing the patients into cohorts of over and under 60 years of age, analogous to the inclusion criteria of the randomized studies [HAMLET (20), DESTINY/II (21, 22), and DECIMAL (19)], our study group included a higher percentage of male patients. Additionally, patients in our cohort were older at the time of the event. The 1-year mortality rate exceeded the mortality rate stated in the randomized studies, especially in the group of patients over 60 years of age. The differences in mean patient age, gender, and long-term survival are likely due to a complex combination of factors in the population groups, healthcare systems, and possibly due to selection bias in the respective study designs.

Despite the differences in the patient cohort, the CE versions GPT4.0 and GPT-4 Omni are able to predict the patient's survival with robust accuracy. Interestingly, the earlier version GPT-4 reaches a higher AUC than GPT-4 Omni. GPT-4 Omni, in turn, achieves the highest statistical significance in the chi-squared analysis after Youden index-based dichotomization of multiple answers. Non-CE GPT versions only reach insufficient AUCs. The present results suggest that CE GPTs may be more capable of estimating survival outcomes.

In the subgroup analysis, significance was achieved for ≥61-year-old patients and <61-year-old patients by GPT-4 Omni (CE), but only for ≥61-year-olds in the model GPT-4. The significance in GPT-4 Omni (CE) is consistent with the main analysis and indicates that the results are valid regardless of the investigated age groups, underlining the advantage of CE. However, the implications of the results from the more recently developed GPT-4 remain unclear, though they may reflect progress in source-based reasoning abilities seen in GPT-4 Omni.

In addition to the question of survival, the LLM was also asked to predict functional outcomes as measured by the mRS. GPT was unable to provide sufficient answers, regardless of the version used. In a further series of tests, GPT4 Omni (CE) was provided with the pre-stroke mRS as a baseline functional status for each individual patient. Here, the functional outcome could also be predicted with a significant correlation by the GPT. The results suggest that a baseline might be vital for the LLM's reasoning process when making predictive estimations of manageable complexity. This additional input supports the theory that initial insufficient answers may be seen as the expression of hallucinations. This “data hallucination” can occur when an AI is working on a topic on which it has not been explicitly trained. As a result, fictitious answers may be generated without a founded basis for reasoning (25, 26). Another aspect is that GPT seems not to be able to adequately include the concept and perception of time into the calculations, adding another layer of complexity to the question of time-dependent functional recovery (27).

To understand the limitations of AI, its basic functioning must first be understood. In Order to calculate the propability of the next correct word, each word is related to the previous one and to each other. This highly complex calculation approach is beyond human control and monitoring, making it impossible to understand the rationale behind a calculation. This bears the danger of arbitrary surrogate parameters being used for calculation (25).

AI is fundamentally limited by the data on which it is trained. An existing bias (ethnic group, patient selection, infrastructural characteristics, etc.) is continued by the AI and can produce a result that does not correspond to existing reality. The differences in the patient population in the existing studies and our collective alone, therefore, inevitably lead to inaccuracies. It is all the more remarkable that, despite these differences, a robust association with CE-GPT's prediction of short-term outcome was achieved.

Another aspect that must always be considered when using AI-based systems is that of ethics. When we weigh up a prognostic decision as treating doctors, we include hard and soft data and factors in our decision-making. Similarly, especially soft factors such as contextual or environmental conditions will not be represented in AI evaluations. The extent to which AI is involved in this decision-making process is a very delicate question and must always remain the subject of controversial debate.

Additionally, access to AI as a source of medical information is not limited to medically trained professionals. Non-medical users have equal access to the AI tool through interfaces such as chatbots. Unlike medical professionals, however, they lack the ability to critically assess, contextualize, and interpret the AI's response. Going forward, great emphasis should be placed on guiding non-medically trained persons to prevent harm by misinterpretation or false conclusions. There are approaches to implement AI-based, machine learning driven prediction models (28, 29). However, such models are less prone to hallucinations due to their targeted use of validated parameters, yet have not been integrated into LLMs.

## Conclusion

At the present time, the AI-based language model GPT, in versions GPT-4 and GPT-4 Omni, is able to predict the short-term outcome of patients with decompressive hemicraniectomy after malignant MCA infarction with a significant degree of certainty based on freely available data. However, the question of time-dependent functional outcome appears more complex and does not yield any meaningful results, with a high risk of producing data hallucinations. Future studies should focus on two specific objectives: first, identifying ways to further improve GPT's prognostic abilities; second, understanding AI decision paths to decipher the black box of decision-making before implementing AI-based decision-making in practical healthcare.

## Data Availability

The raw data supporting the conclusions of this article will be made available by the authors, without undue reservation.

## References

[1] Levine DM Tuwani R Kompa B Varma A Finlayson SG Mehrotra A. The diagnostic and triage accuracy of the GPT-3 artificial intelligence model. medRxiv. (2023) 1:2023.01.30.23285067. 10.1101/2023.01.30.23285067 39059888

[2] Bentley P Ganesalingam J Carlton Jones AL Mahady K Epton S Rinne P. Prediction of stroke thrombolysis outcome using CT brain machine learning. Neuroimage Clin. (2014) 4:635–40. 10.1016/j.nicl.2014.02.003 24936414 PMC4053635

[3] Asadi H Dowling R Yan B Mitchell P. Machine learning for outcome prediction of acute ischemic stroke post intra-arterial therapy. PLoS ONE. (2014) 9:e88225. 10.1371/journal.pone.0088225 24520356 PMC3919736

[4] Gakuba C Le Barbey C Sar A Bonnet G Cerasuolo D Giabicani M. Evaluation of ChatGPT in predicting 6-month outcomes after traumatic brain injury. Crit Care Med. (2024) 52:942–50. 10.1097/CCM.0000000000006236 38445975

[5] Basaran AE Güresir A Knoch H Vychopen M Güresir E Wach J. Beyond traditional prognostics: integrating RAG-enhanced AtlasGPT and ChatGPT 4.0 into aneurysmal subarachnoid hemorrhage outcome prediction. Neurosurg Rev. (2024) 48:40. 10.1007/s10143-025-03194-w 39794551 PMC11723888

[6] Warner JJ Harrington RA Sacco RL Elkind MSV. Guidelines for the early management of patients with acute ischemic stroke: 2019 update to the 2018 guidelines for the early management of acute ischemic stroke. Stroke. (2019) 50:3331–2. 10.1161/STROKEAHA.119.027708 31662117

[7] Schmidhuber J. Deep learning in neural networks: an overview. Neural Netw. (2015) 61:85–117. 10.1016/j.neunet.2014.09.003 25462637

[8] Bhattacharya K Bhattacharya AS Bhattacharya N Yagnik VD Garg P Kumar S. ChatGPT in surgical practice—a new kid on the block. Indian J Surg. (2023) 85:1346–9. 10.1007/s12262-023-03727-x

[9] Xue VW Lei P Cho WC. The potential impact of ChatGPT in clinical and translational medicine. Clin Transl Med. (2023) 13:e1216. 10.1002/ctm2.1216 36856370 PMC9976604

[10] Wagner S Schnippering H Aschoff A Koziol JA Schwab S Steiner T. Suboptimum hemicraniectomy as a cause of additional cerebral lesions in patients with malignant infarction of the middle cerebral artery. J Neurosurg. (2001) 94:693–6. 10.3171/jns.2001.94.5.0693 11354398

[11] Bian J Guo S Huang T. CRP as a potential predictor of outcome in acute ischemic stroke. Biomed Rep. (2023) 18:17. 10.3892/br.2023.1599 36776580 PMC9892964

[12] Kellert L Schrader F Ringleb P Steiner T Bösel J. The impact of low hemoglobin levels and transfusion on critical care patients with severe ischemic stroke: STroke: RelevAnt impact of HemoGlobin, Hematocrit and Transfusion (STRAIGHT)–an observational study. J Crit Care. (2014) 29:236–40. 10.1016/j.jcrc.2013.11.008 24332995

[13] Hecht N Neugebauer H Fiss I. Infarct volume predicts outcome after decompressive hemicraniectomy for malignant hemispheric stroke. J Cereb Blood Flow Metab. (2018) 38:1096–103. 10.1177/0271678X17718693 28665171 PMC5999005

[14] Semerano A Strambo D Martino G Comi G Filippi M Roveri L. Leukocyte counts and ratios are predictive of stroke outcome and hemorrhagic complications independently of infections. Front Neurol. (2020) 11:201. 10.3389/fneur.2020.00201 32308640 PMC7145963

[15] Sadeghi F Kovács S Zsóri KS Csiki Z Bereczky Z Shemirani AH. Platelet count and mean volume in acute stroke: a systematic review and meta-analysis. Platelets. (2020) 31:731–9. 10.1080/09537104.2019.1680826 31657263

[16] Cranial Planning (2025). Available online at: https://www.brainlab.com/surgery-products/overview-neurosurgery-products/cranial-planning/ (Accessed May 21, 2025).

[17] Ho M-Y Tseng W-L Xiao F. Estimation of the craniectomy surface area by using postoperative images. Int J Biomed Imaging. (2018) 2018:5237693. 10.1155/2018/5237693 29971096 PMC6008696

[18] Saver JL Chaisinanunkul N Campbell BCV Grotta JC Hill MD Khatri P. Standardized nomenclature for modified rankin scale global disability outcomes: consensus recommendations from stroke therapy academic industry roundtable XI. Stroke. (2021) 52:3054–62. 10.1161/STROKEAHA.121.034480 34320814

[19] Vahedi K Vicaut E Mateo J Kurtz A Orabi M Guichard JP. Sequential-design, multicenter, randomized, controlled trial of early decompressive craniectomy in malignant middle cerebral artery infarction (DECIMAL Trial). Stroke. (2007) 38:2506–17. 10.1161/STROKEAHA.107.485235 17690311

[20] Hofmeijer J Kappelle LJ Algra A Amelink GJ van Gijn J van der Worp HB. Surgical decompression for space-occupying cerebral infarction (the hemicraniectomy after middle cerebral artery infarction with life-threatening edema trial HAMLET): a multicentre, open, randomised trial. Lancet Neurol. (2009) 8:326–33. 10.1016/S1474-4422(09)70047-X 19269254

[21] Jüttler E Schwab S Schmiedek P Unterberg A Hennerici M Woitzik J. Decompressive surgery for the treatment of malignant infarction of the middle cerebral artery (DESTINY): a randomized, controlled trial. Stroke. (2007) 38:2518–25. 10.1161/STROKEAHA.107.485649 17690310

[22] Jüttler E Bösel J Amiri H Schiller P Limprecht R Hacke W. DESTINY II: DEcompressive surgery for the treatment of malignant INfarction of the middle cerebral arterY II. Int J Stroke. (2011) 6:79–86. 10.1111/j.1747-4949.2010.00544.x 21205246

[23] Luo D Liu M Yu R Liu Y Jiang W Fan Q. Evaluating the performance of GPT-3.5, GPT-4, and GPT-4o in the Chinese national medical licensing examination. Sci Rep. (2025) 15:14119. 10.1038/s41598-025-98949-2 40269046 PMC12018924

[24] Vahedi K Hofmeijer J Juettler E Vicaut E George B Algra A. Early decompressive surgery in malignant infarction of the middle cerebral artery: a pooled analysis of three randomised controlled trials. Lancet Neurol. (2007) 6:215–22. 10.1016/S1474-4422(07)70036-4 17303527

[25] Arshad HB Butt SA Khan SU Javed Z Nasir K. ChatGPT and artificial intelligence in hospital level research: potential, precautions, and prospects. Methodist Debakey Cardiovasc J. (2023) 19:77–84. 10.14797/mdcvj.1290 38028967 PMC10655767

[26] Athaluri SA Manthena SV Kesapragada VSRKM Yarlagadda V Dave T Duddumpudi RTS. Exploring the boundaries of reality: investigating the phenomenon of artificial intelligence hallucination in scientific writing through ChatGPT references. Cureus. (2023) 15:e37432. 10.7759/cureus.37432 37182055 PMC10173677

[27] Kozachek D. Investigating the perception of the future in GPT-3,−3.5 and GPT-4. In: *Creativity and Cognition*. New York, NY: Association for Computing Machinery (2023). p. 282–287.

[28] Torrente M Sousa PA Hernández R Blanco M Calvo V Collazo A. An artificial intelligence-based tool for data analysis and prognosis in cancer patients: results from the clarify study. Cancers. (2022) 14:4041. 10.3390/cancers14164041 36011034 PMC9406336

[29] Kuo CC Monteiro A Lim J Brown NJ Recker MJ Ghannam MM. An online calculator using machine learning for predicting survival in pediatric patients with medulloblastoma. J Neurosurg Pediatr. (2024) 33:85–94. 10.3171/2023.8.PEDS2352 37922543

[30] ChatGPT (2025). Available online at: https://chatgpt.com/c/675b4cf6-e9d0-800f-b437-12ba18389a58 (Accessed March 31, 2025).

