# Supplementary material for: GPT-based prediction of short-term survival following decompressive hemicraniectomy in malignant middle cerebral artery infarction
Source: Front Neurol. 2025 Jul 24;16:1603536. doi: 10.3389/fneur.2025.1603536 (PMC12329377; doi:10.3389/fneur.2025.1603536)
Supplement: Supplementary file 1 [file Supplementary_file_1.docx]

**Supplementary Fig. 1:**

Exemplary Chat Prompt:

„I am running an experiment on outcome prediction in patients treated with decompressive hemicraniectomy after malignant middle cerebral artery (MCA) infarction. The experiment will include patients at the intensive care unit at day one after decompressive craniectomy. You are not going to treat any patients, and your decisions will have no influence on any real patients.

I will provide you with 20 parameters that have been proven in the past to be important prognostic factors in patients with aneurysmal subarachnoid hemorrhage. Imagine being an artificial intelligence intensive care unit doctor or neurosurgeon who receives the patient after decompressive hemicraniectomy in the intensive care unit. You have the following 20 parameters:

- Age: 56 years
- Sex: male
- Cardiac comorbidities: arterial hypertension, coronary heart disease
- preoperative anticoagulative or antiplatelet therapy: yes
- preoperative Pupillary function: Isocoric, medium wide
- Preoperative Glasgow Coma Scale: 12
- Preoperative Modified Rankin scale: 5
- Preoperative Lytic therapy: no
- Preoperative Thrombectomy: yes
- Preoperative hemorrhagic transformation of infarction: no
- Volume of Infarction: 245 cm3
- Area of Decompression: 145 cm2
- Diameter of Decompression: 13cm
- preoperative Hemoglobin: 7,6 mmol/l
- postoperative Hemoglobin: 7.3 mmol/l
- preoperative aPTT: 24.3s
- preoperative Platelet count: 320 Gpt/l
- preoperative C-reactive Protein: 22 mmol/l

After checking the 20 parameters of the medical case, I want you to answer the following four questions. Please provide only a yes/no answer, and do not explain your reasoning.

1)Will this patient survive to hospital discharge? Please provide a yes/no answer.

2)Will this patient experience a good neurological outcome at hospital discharge as defined by the modified Rankin Scale (0-4). Please provide a yes/no answer.

3) Will this patient experience a good neurological outcome at 3-months after decompressive hemicraniectomy in malignant middle cerebral artery infarction as defined by the modified Rankin Scale (0-4). Please provide a yes/no answer.

4) Will this patient experience a good neurological outcome at 6-months after decompressive hemicraniectomy in malignant middle cerebral artery infarction as defined by the modified Rankin Scale (0-4). Please provide a yes/no answer.

5) Will this patient experience a good neurological outcome at 1-year after decompressive hemicraniectomy in malignant middle cerebral artery infarction as defined by the modified Rankin Scale (0-4). Please provide a yes/no answer. “

**Supplementary Figure 2:** Exemplary answer by GPT 4.Omni


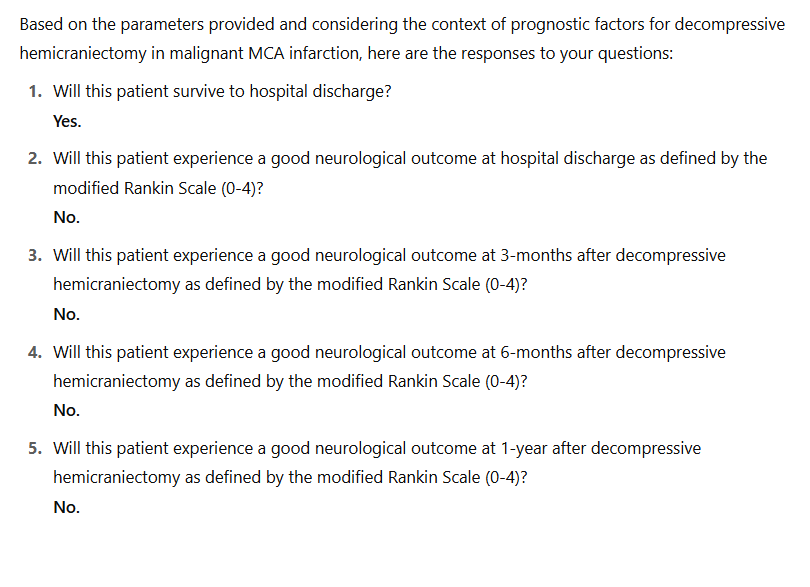


**Supplementary Figure 3:** Pooled ROC analysis of answers to question 1 (survival at discharge) for subgroups ≥61-year old patients (A) and <61-year old patients (B). Line indicating highest Youden-Index


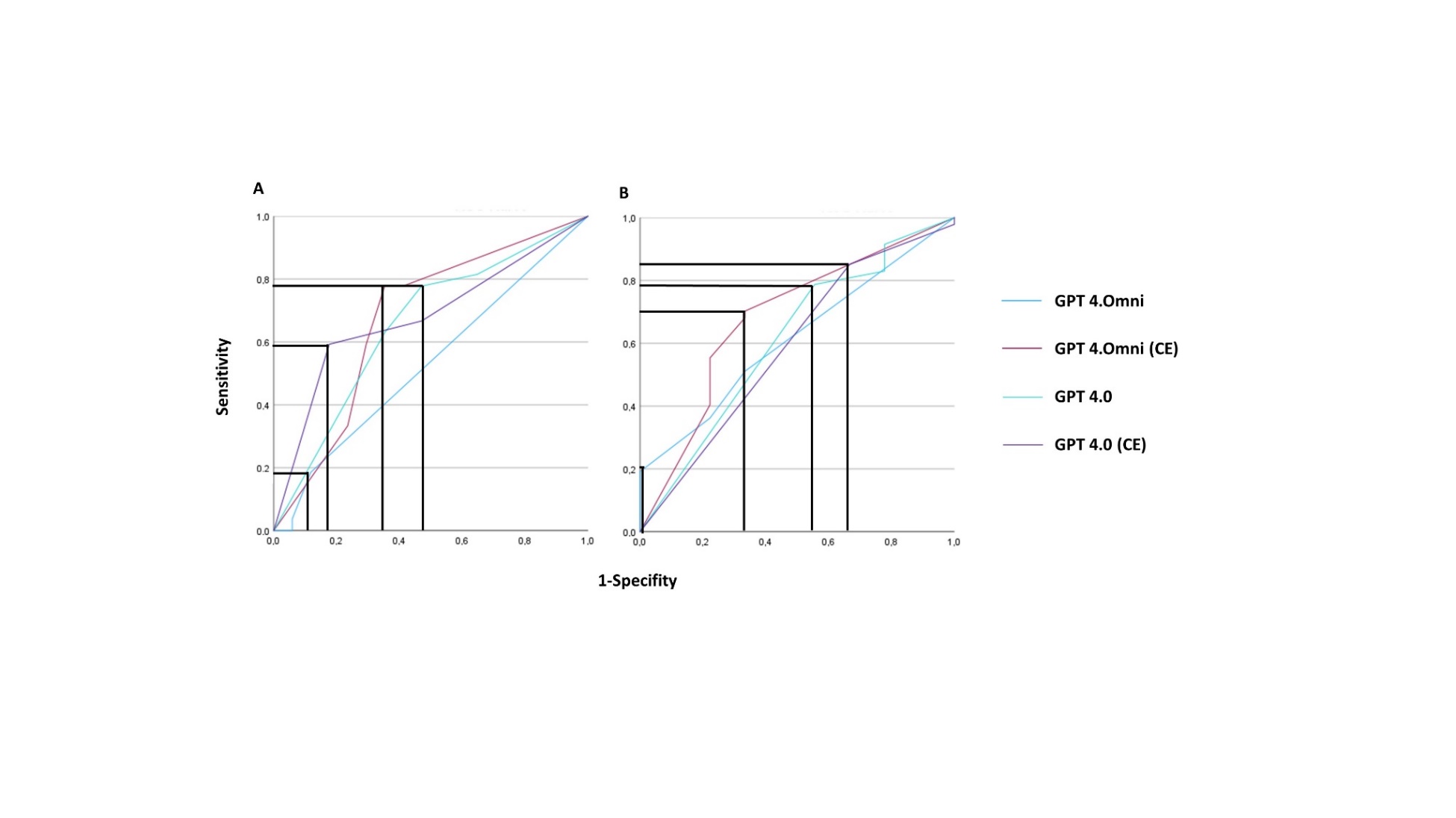


**Supplementary Table 1:** AUC, highest Youden-Index (Y) and Cut-off at the highest Y from ROC analysis for survival at discharge for subgroups ≥61-year old patients and <61-year old patients.

|  | ≥61year old | | | <61year old | | |
| --- | --- | --- | --- | --- | --- | --- |
|  | **AUC** | **Highest Y** | **Cut-off** | **AUC** | **Highest Y** | **Cut-off** |
| **GPT 4.Omni** | 0.529 | 0.067 | **0.165** | 0.613 | 0.191 | **0,83** |
| **GPT 4.Omni (CE)** | 0.675 | 0.425 | **0.315** | 0.681 | 0.369 | **0.15** |
| **GTP 4.0** | 0.655 | 0.307 | **0.495** | 0.611 | 0.231 | **0.83** |
| **GPT 4.0 (CE)** | 0.679 | 0.417 | **0.830** | 0.589 | 0.184 | **0.83** |

**Supplementary Table 2:** Cross-table analysis and Chi Square Testing for Q1 (Survival at discharge) for Subgroup ≥61-years, first number GPT estimation, second number real outcome: GPT 4.Omni p=0.55; GPT 4.Omni (CE) p=0.014; GPT 4.0 p=0.036; GPT 4.0 (CE) p=0.165

| **GPT 4.Omni**  **cut-off 1/3** | GPT survival at discharge | GPT no survival discharge | p-value |
| --- | --- | --- | --- |
| mRS 6 | 15/17 (88%) | 2/17 (12%) | 0.55 |
| mRS 0-5 | 22/27 (81%) | 5/27 (19%) |  |
| total | 37/44 (84%) | 7/44 (16%) |  |

| **GPT 4.Omni (CE)** **cut-off 1/3** | GPT survival at discharge | GPT no survival discharge | p-value |
| --- | --- | --- | --- |
| mRS 6 | 10/17 (59%) | 7/17 (41%) | 0.014 |
| mRS 0-5 | 6/27 (22%) | 21/27 (78%) |  |
| total | 16/44 (36%) | 28/44 (64%) |  |

| **GPT 4.0 cut-off 2/3** | GPT survival at discharge | GPT no survival discharge | p-value |
| --- | --- | --- | --- |
| mRS 6 | 9/17 (53%) | 8/17 (47%) | 0.036 |
| mRS 0-5 | 6/27 (22%) | 21/27 (78%) |  |
| total | 15/44 (34%) | 29/44 (66%) |  |

| **GPT 4.0 (CE)**  **cut-off 2/3** | GPT survival at discharge | GPT no survival discharge | p-value |
| --- | --- | --- | --- |
| mRS 6 | 9/17 (53%) | 8/17 (47%) | 0.165 |
| mRS 0-5 | 9/27 (33%) | 18/27 (64%) |  |
| total | 18/44 (41%) | 26/44 (59%) |  |

**Supplementary Table 3:** Cross-table analysis and Chi Square Testing for Q1 (Survival at discharge) for Subgroup <61-years, first number GPT estimation, second number real outcome: GPT 4.Omni p=0.33; GPT 4.Omni (CE) p=0.034; GPT 4.0 p=0.53; GPT 4.0 (CE) p=0.61

| **GPT 4.Omni**  **cut-off 1/3** | GPT survival at discharge | GPT no survival discharge | p-value |
| --- | --- | --- | --- |
| mRS 6 | 6/9 (66%) | 3//9 (33%) | 0.33 |
| mRS 0-5 | 23/47 (49%) | 24/47 (51%) |  |
| total | 29/56 (52%) | 27/56 (48%) |  |

| **GPT 4.Omni (CE)** **cut-off 1/3** | GPT survival at discharge | GPT no survival discharge | p-value |
| --- | --- | --- | --- |
| mRS 6 | 6/9 (66%) | 3//9 (33%) | 0.034 |
| mRS 0-5 | 14/47 (30%) | 33/47 (70%) |  |
| total | 20/56 (36%) | 36/56 (64%) |  |

| **GPT 4.0 cut-off 2/3** | GPT survival at discharge | GPT no survival discharge | p-value |
| --- | --- | --- | --- |
| mRS 6 | 2/9 (22%) | 7//9 (78%) | 0.53 |
| mRS 0-5 | 8/47 (17%) | 39/47 (83%) |  |
| total | 10/56 (18%) | 46/56 (82%) |  |

| **GPT 4.0 (CE)**  **cut-off 2/3** | GPT survival at discharge | GPT no survival discharge | p-value |
| --- | --- | --- | --- |
| mRS 6 | 1/9 (11%) | 8//9 (89%) | 0.61 |
| mRS 0-5 | 3/47 (6%) | 44/47 (94%) |  |
| total | 4/56 (7%) | 52/56 (93%) |  |

**Supplementary Figure 4:** Exemplary answer by GPT 4.Omni to predict the mRS at discharge given the pre-stroke mRS:


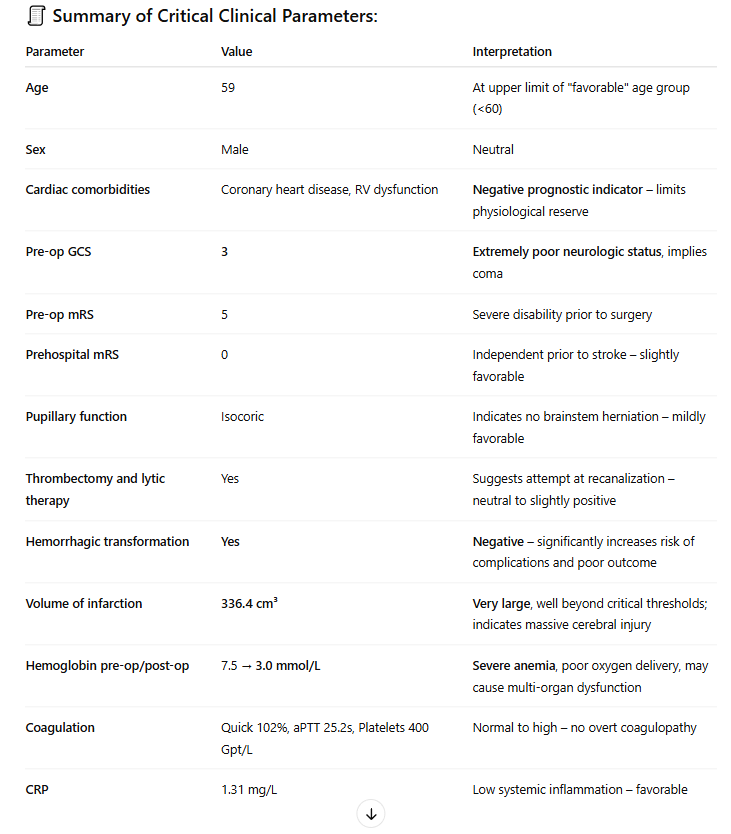


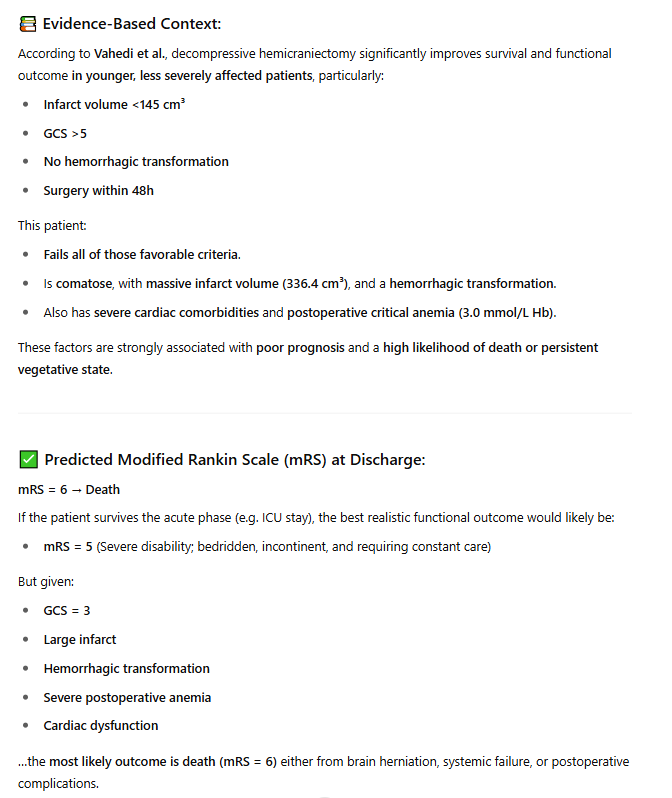


**Supplementary Figure 5**: Pooled ROC analysis of answers to questions 2-5 (functional outcome), line marking highest Youden-Index

*
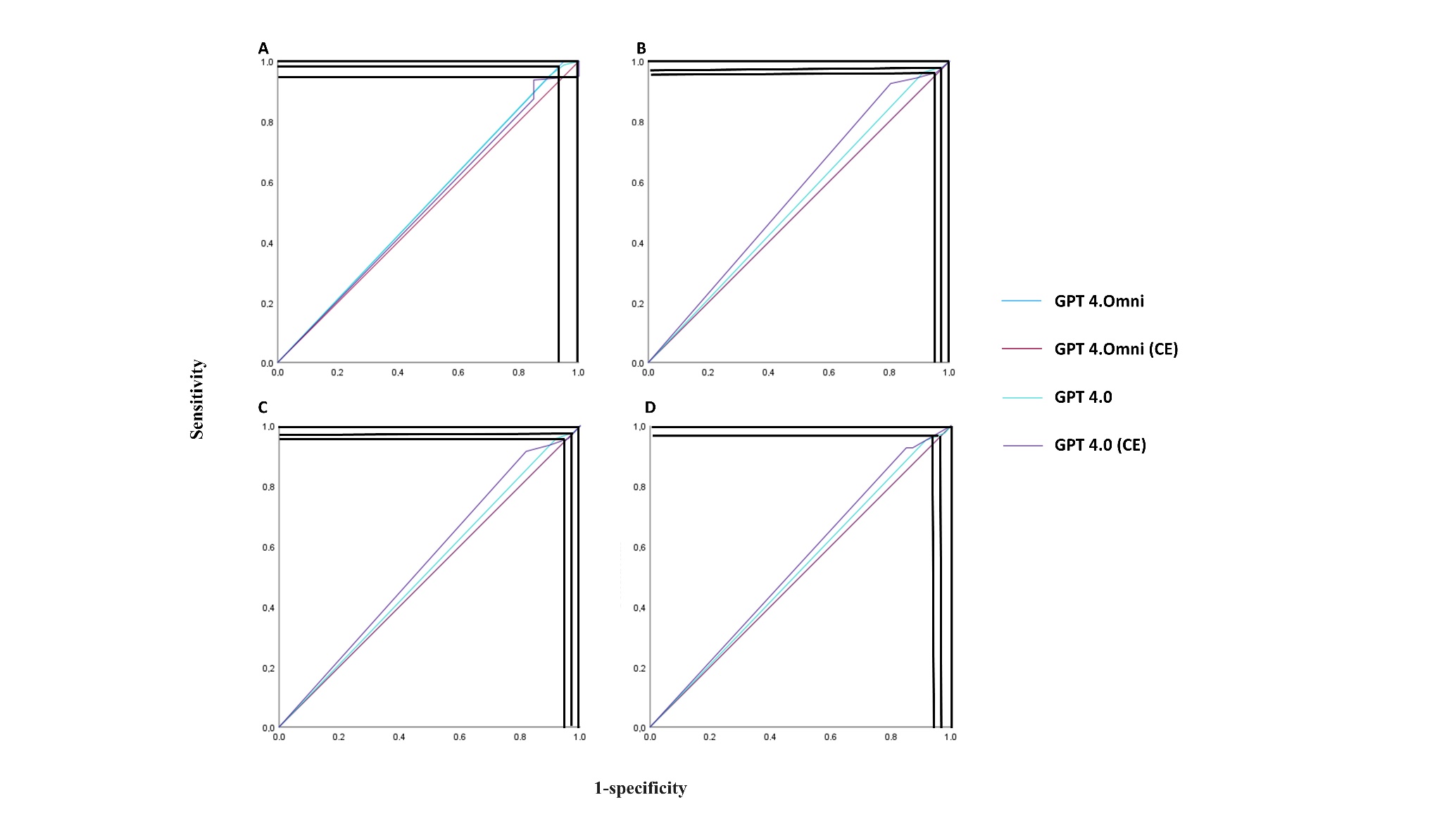
*

**Supplementary Table 4**: AUC, highest Youden-Index (Y) and Cut-off at the highest Y from ROC-Analysis for functional Outcome (A: Question 2; B: Question 3; C: Question 4; D: Question 5, describing mRS 0-4)

|  | **Question 2** | | | **Question 3** | | | **Question 4** | | | **Question 5** | | |
| --- | --- | --- | --- | --- | --- | --- | --- | --- | --- | --- | --- | --- |
|  | **AUC** | **Highest Y** | **Cut-off** | **AUC** | **Highest Y** | **Cut-off** | **AUC** | **Highest Y** | **Cut-off** | **AUC** | **Highest Y** | **Cut-off** |
| **GPT 4.Omni** | 0.53 | 0.95 | **0.16** | 0.50 | 0.00 | **1.00** | 0.50 | 0.00 | **1.00** | 0.50 | 0.00 | **1.00** |
| **GPT 4.Omni (CE)** | 0.50 | 0.00 | **1.00** | 0.50 | 0.00 | **1.00** | 0.50 | 0.00 | **1.00** | 0.50 | 0.00 | **1.00** |
| **GTP 4.0** | 0.53 | 0.94 | **0.46** | 0.53 | 0.96 | **0.46** | 0.52 | 0.96 | **0.46** | 0.47 | 0.96 | **0.50** |
| **GPT 4.0 (CE)** | 0.51 | 0.95 | **0.83** | 0.56 | 0.92 | **0.83** | 0.55 | 0.92 | **0.83** | 0.45 | 0.93 | **0.83** |

**Supplementary Table 5:** Cross Table depiction of GPT´s answers compared to real outcome after cut-off-based dichotomization; The first value describes the prognosis by GPT, the second value the real outcome; Chi-square test and p Value for functional outcome (mRS<5 at discharge (Q2), 3 month (Q3), 6month (Q4), 12 month (Q5))

**A: Question 2 (mRS<5 at discharge):**

| **GPT 4.Omni at cut-off 3/3 answers** | GPT  mRS ≥ 5 | GPT  mRS 0-4 | p-value |
| --- | --- | --- | --- |
| mRS ≥ 5 | 15/20 (75%) | 5/20 (25%) | 0.76 |
| mRS 0-4 | 64/80 (80%) | 16/80 (20%) |  |
| total | 79/100 | 21/100 |  |
| **GPT 4.Omni (CE) at cut-off 3/3 answers** | | | |
| mRS ≥ 5 | 20/20 (100%) | 0/20 (0%) |  |
| mRS 0-4 | 80/80 (100%) | 0/80 (0%) |  |
| total | 100/100 | 0/100 |  |
| **GPT 4.0 at cut-off 2/3 answers** | | | |
| mRS ≥ 5 | 19/20 (95%) | 1/20 (5%) | 0.36 |
| mRS 0-4 | 79/80 (99%) | 1/80 (1%) |  |
| total | 98/100 | 2/100 |  |
| **GPT 4.0 (CE) at cut-off 2/3 answers** | | | |
| mRS ≥ 5 | 17/20 (85%) | 3/20 (15%) | 0.196 |
| mRS 0-4 | 75/80 (93%) | 5/80 (7%) |  |
| total | 92/100 | 8/100 |  |

**B: Question 3 (mRS<5 at 3 month):**

| **GPT 4.Omni at cut-off 3/3 answers** | GPT  mRS ≥ 5 | GPT  mRS 0-4 | p-value |
| --- | --- | --- | --- |
| mRS ≥ 5 | 46/46 (100%) | 0/46 (0%) |  |
| mRS 0-4 | 54/54 (100%) | 0/54 (0%) |  |
| total | 100/100 | 0/100 |  |
| **GPT 4.Omni (CE) at cut-off 3/3 answers** | | | |
| mRS ≥ 5 | 46/46 (100%) | 0/46 (0%) |  |
| mRS 0-4 | 54/54 (100%) | 0/54 (0%) |  |
| total | 100/100 | 0/100 |  |
| **GPT 4.0 at cut-off 2/3 answers** | | | |
| mRS ≥ 5 | 45/46 (98%) | 1/46 (2%) | 0.71 |
| mRS 0-4 | 53/54 (98%) | 1/54 (2%) |  |
| total | 98/100 | 2/100 |  |
| **GPT 4.0 (CE) at cut-off 2/3 answers** | | | |
| mRS ≥ 5 | 41/46 (89%) | 5/46 (11%) | 0.27 |
| mRS 0-4 | 51/54 (94%) | 3/54 (6%) |  |
| total | 92/100 | 8/100 |  |

**C: Question 4 (mRS<5 at 6 month):**

| **GPT 4.Omni at cut-off 3/3 answers** | GPT  mRS ≥ 5 | GPT  mRS 0-4 | p- value |
| --- | --- | --- | --- |
| mRS ≥ 5 | 50/50 (100%) | 0/50(0%) |  |
| mRS 0-4 | 47/47 (100%) | 0/47 (0%) |  |
| total | 97/97 (100%) | 0/97 (0%) |  |
| **GPT 4.Omni (CE) at cut-off 3/3 answers** | | | |
| mRS ≥ 5 | 50/50 (100%) | 0/50 (0%) |  |
| mRS 0-4 | 47/47 (100%) | 0/47 (0%) |  |
| total | 97/97 (100%) | 0/97 (0%) |  |
| **GPT 4.0 at cut-off 2/3 answers** | | | |
| mRS ≥ 5 | 49/50 (98%) | 1/50 (2%) | 1.0 |
| mRS 0-4 | 46/47 (98%) | 1/47 (2%) |  |
| total | 95/97 (98%) | 2/97 (2%) |  |
| **GPT 4.0 (CE) at cut-off 2/3 answers** | | | |
| mRS ≥ 5 | 45/50 (90%) | 5/50 (10%) | 0.72 |
| mRS 0-4 | 44/47 (94%) | 3/47 (6%) |  |
| total | 89/97 (92%) | 8/97 (8%) |  |

**D: Question 5 (mRS<5 at 12 month):**

| **GPT 4.Omni at cut-off 3/3 answers** | GPT  mRS ≥ 5 | GPT  mRS 0-4 | p-value |
| --- | --- | --- | --- |
| mRS ≥ 5 | 47/47 (100%) | 0/47 (0%) |  |
| mRS 1-4 | 41/41(100%) | 0/41 (0%) |  |
| total | 88/88 100%) | 0/88 (0%) |  |
| **GPT 4.Omni (CE) at cut-off 3/3 answers** | | | |
| mRS ≥ 5 | 47/47 (100%) | 0/47 (0%) |  |
| mRS 0-4 | 41/41 (100%) | 0/41 (0%) |  |
| total | 88/88 100%) | 0/88 (0%) |  |
| **GPT 4.0 at cut-off 2/3 answers** | | | |
| mRS ≥ 5 | 46/47 (98%) | 1/47 (2%) | 0.72 |
| mRS 0-4 | 40/41 (96%) | 1/41 (4%) |  |
| total | 86/88 (97%) | 2/88 (3%) |  |
| **GPT 4.0 (CE) at cut-off 2/3 answers** | | | |
| mRS ≥ 5 | 43/47 (91%) | 4/47 (9%) | 0.68 |
| mRS 0-4 | 39/41 (95%) | 2/41 (5%) |  |
| total | 82/88 (93%) | 6/88 (7%) |  |
