# Supplementary figures and images for: GPT-based prediction of short-term survival following decompressive hemicraniectomy in malignant middle cerebral artery infarction
Source: Front Neurol. 2025 Jul 24;16:1603536. doi: 10.3389/fneur.2025.1603536 (PMC12329377; doi:10.3389/fneur.2025.1603536)

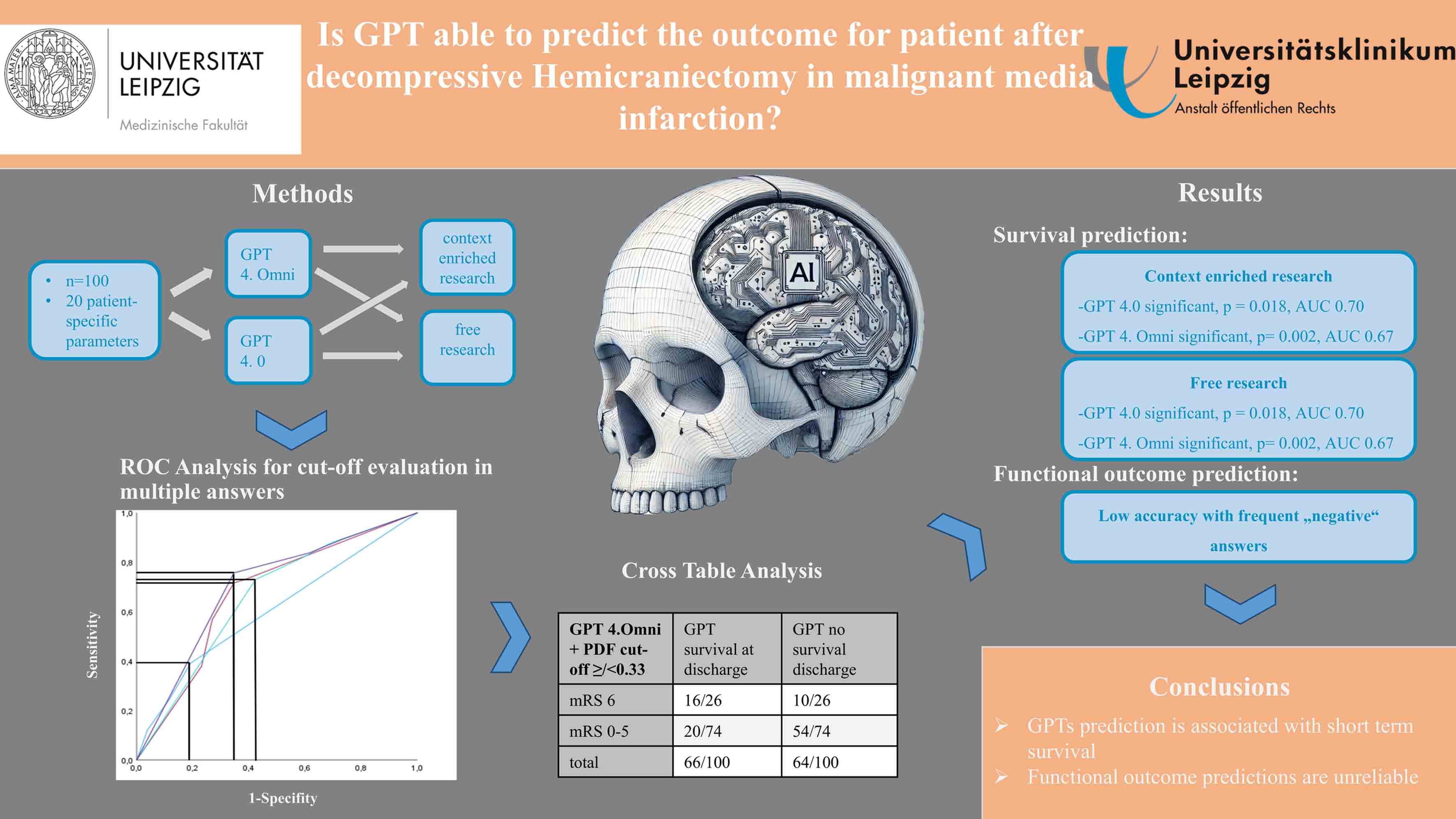

Supplement: Supplementary file 2 [file Image_1.jpeg]
